# Iatrogenic Chymobilia— A Disease of the Nineties?

**DOI:** 10.1155/1991/26202

**Published:** 1991

**Authors:** George W. Johnston

**Affiliations:** Department of Surgery Royal Victoria Hospital Grosvenor Road Belfast BT12 6BA UK


*HPB Surgery*, 1991, Vol. 4, pp. 187-190
Reprints available directly from the publisher
Photocopying permitted by license only

© 1991 Harwood Academic Publishers GmbH
Printed in the United Kingdom

### IATROGENIC CHYMOBILIA — A DISEASE OF THE NINETIES?

GEORGE W. JOHNSTON

*Department of Surgery, Royal Victoria Hospital, Grosvenor Road, Belfast BT12 6BA, UK*

*(Received 18 February 1991)*

The last two decades have seen a strong swing towards preservation of some of the sphincters of the alimentary tract, namely, the pyloric and anal sphincters, while at the same time there has been increasing "vandalism" of the sphincter at the distal end of the common bile duct.

Endoscopic papillotomy, first introduced in 1974, is a very useful procedure in selected patients but too liberal use of the technique may result in a harvest of new problems in years to come. The intact sphincter of Oddi prevents the entry of duodenal contents into the biliary tree; its division allows their free reflux. By the term 'chymobilia' we mean the presence of chyme in the biliary tree. The Greek word chyme (juice) is combined with the Latin word bilis (bile) in a manner similar to the term haemobilia introduced by Sandblom in 1948[1]. Following endoscopic sphincterotomy reflux of duodenal chyme occurs in most patients; aerobilia is seen in half and bacterobilia in all[2]. The results of surgical sphincterotomy are similar. The majority of these patients with bacterobilia do not develop classical symptoms of ascending cholangitis, namely pain, pyrexia, rigors and jaundice but 20 per cent have bouts of upper abdominal pain and associated elevation of the serum Gamma glutamyltranspepside[3]. Following biliary-enteric anastomosis both aerobic and anaerobic bacteria are involved[4]. Bacterobilia may not be as innocent as was initially thought, and for patients with immunosuppression, the risk of cholangitis is markedly increased[5]. In addition the reflux of chyme may set up chemical changes with resultant inflammatory reaction in the duct epithelium and periductal area. The clinical syndrome of ascending cholangitis often indicates outflow obstruction usually of an incomplete nature due to residual or recurrent stones or restenosis. However we have seen cholangitis in patients following sphincterotomy and other types of biliary-enteric anastomosis where a free-flowing, unobstructed biliary tree was demonstrated by percutaneous cholangiography and by retrograde barium and air studies of the ductal system.

Goldman and colleagues reported on six patients who suffered repeated episodes of cholangitis despite widely patent biliary-enteric anastomoses[6]. Debris is frequently seen in the duct following fenestration whether it be choledochoduodenostomy where the sump syndrome can occur, or after simple surgical or endoscopic sphincterotomy. These ductal filling defects are sometimes referred to as false calculi images. Escourrou and colleagues found evidence of reflux from the duodenum into the biliary tree in 65 per cent of patients after endoscopic sphincterotomy but state that they never observed clinical symptoms related to reflux in the absence of recurrent or retained stones[7]. However the authors did not give any results of liver function studies in their patients following sphincterotomy. The reflux of chemical and bacterial irritants is probably a major factor in the production of the elevated serum bilirubin and hepatic enzymes seen in 36 per cent of patients with choledochoduodenostomy and 17 per cent of patients with transduodenal sphincterotomy as noted in a surgical series of 246 biliary fenestrations reported from Leicester[8].

Although reflux may not always produce a clinical syndrome, the biochemical changes indicate at least some degree of continuing low grade damage within the liver parenchyma. Greenfield and colleagues found periportal fibrosis and inflammation on liver biopsy in four out of five patients with raised transaminase levels[3]. Eleftheriadis and others biopsied the bile duct mucosa one to twelve years after choledochoduodenostomy in nine asymptomatic patients. In all nine patients they found hyperplasia of the epithelial cells, metaplastic goblet cells, pseudopyloric gland formation, dense inflammatory cell infiltration with lymphocytes, plasma cells and polymorphonuclear cells and also fibrosis[9]. Not surprisingly, similar findings were noted in an experimental study using sphincterotomy instead of choledochoduodenostomy[10]. Some consider that the changes in the main biliary ducts are adaptive and therefore beneficial. However the liver biopsies show that even the smaller ducts suffer from this inflammatory reaction. Fibrosis and inflammation in these small calibre ducts is likely to cause obstruction, biliary stasis and ultimately parenchymal change. My contention is that iatrogenic chymobilia with its accompanying bacterobilia can produce secondary sclerosing cholangitis in the absence of any obstructive element. The reason that we have not seen a lot of secondary cholangitis and secondary biliary cirrhosis in the past is that many of the surgically created biliary-enteric anastomoses or sphincterotomies were performed in elderly patients with recurrent stones or as bypass procedures in patients with malignancy, both groups having relatively short life expectancy. However much younger patients with benign disease are now being subjected to endoscopic sphincterotomies and I believe that we will see a crop of problems secondary to chymobilia in the decade in which we have entered and subsequently.

Particularly worrying is the use of sphincterotomy for ductal stones without cholecystectomy in some relatively young patients. The residual gallbladder can act as a focus of infection following the ascent of bacteria from the gut. Certainly choledochoduodenostomy without cholecystectomy in the dog results in inevitable chronic inflammation of the gallbladder with the formation of concretions in two thirds of the animals[11]. Even a Roux-en-Y anastomosis does not protect the dog from ascending cholangitis if the gallbladder is left *in situ* during biliary-enteric anastomosis. Safraney and colleagues noted that acute cholecystitis occured in 11 per cent of patients subjected to endoscopic sphincterotomy without cholecystectomy[13]. When doing surgery for benign disease of the biliary tree surgeons often go to great lengths to try and prevent reflux of chyme. Creation of valved conduits, interposed jejunal segments and Roux-en-Y procedures have all been used[14,15] but the outcome is not always satisfactory. The Roux-en-Y operation which was first introduced for gastric surgery using a 20 cm limb has been adapted for biliary-enteric anastomoses[16]. Initially the length of the Roux limb which was advocated in biliary surgery was quite short and it has been fascinating to see the "lengthening Roux-en-Y syndrome" (Table 1). Although the currently recommended length is 70 cm, we have demonstrated reflux of oral barium into the biliary

**Table 1** The lengthening roux-en-y limb

| *Author* | *Length in cm* | *Year* |
|---|---|---|
| Smith | 20 | 1964 |
| Thorbjarnarson | 25 | 1975 |
| Warren | 30 | 1980 |
| Takahasi | 35 | 1988 |
| Kune | 40 | 1980 |
| Gadacz | 60 | 1983 |
| Bismuth | 70 | 1978 |
| Blumgart | 70 | 1988 |
| Who Knows? | 90 | 2000 |

(References 17–24)

tree with 70 cm Roux-en-Y limbs. Perhaps an 80 cm or longer Roux-en-Y limb is required to prevent reflux of orally ingested material. This may reduce the incidence of clinically significant ascending cholangitis but can not stop chymobilia. Once the sphincter mechanism is destroyed nothing prevents the potentially dangerous reflux of bowel contents into the unprotected biliary tree. When Dr Summerfield argued that biliary obstruction is best managed by endoscopists, he wisely added, "Probably only a proportion of the new techniques will stand the test of time!" Perhaps we clinicians need to exercise more caution if we are to avoid the potential hazards of chymobilia.

### *References*

1. Sandblom, P. (1948) Haemorrhage into the biliary tract following trauma — traumatic hemobilia. *Surgery*, **94**, 271
2. Seifert, E. (1988) Long term follow up after endoscopic sphincterotomy (EST). *Endoscopy*, **20**, 232–235
3. Greenfield, C., Clelland, P., Dick, R., Masters, S., Summerfield, J.A. and Sherlock, S. (1985) Biliary sequalae of endoscopic sphincterotomy. *Postgraduate Medical Journal*, **61**, 213–215
4. Brook, I. and Altman, R.P. (1984) The significance of anaerobic bacteria in biliary tract infection after hepatic porto-enterostomy for biliary atresia. *Surgery*, **95**, 281–283
5. Sharma, B.K., Pounder, R.E., Kirk, R.M., Noone, P. and Wilson, D.W. (1985) Disseminated infection associated with cortico-steroid therapy after transduodenal sphincterotomy. *Journal of Infection*, **10**, 60–64
6. Goldmann, L.D., Steer, M.L. and Silen, W. (1983) Recurrent cholangitis after biliary surgery. *American Journal of Surgery*, **145**, 450–454
7. Escourrou, J., Cordova, J.A., Lazorthes, F., Frexinos, J. and Ribet, A. (1984) Early and late complications after endoscopic sphincterotomy for biliary lithiasis with and without the gallbladder in situ. *GUT*, **25**, 598–602
8. Baker, A.R., Neoptolemos, J.P., Leese, T., James, D.C. and Fossard, D.P. (1987) Long term follow up of patients with side to side choledochoduodenostomy and transduodenal sphincterotomy. *Annals of the Royal College of Surgeons of England*, **69**, 253–258
9. Eleftheriadis, E., Tzioufa, V., Kotzampassi, K. and Aletras, H. (1988) Common bile duct mucosa in choledochoduodenostomy patients — histological and histochemical study. *HPB Surgery*, **1**, 15–20
10. Fihlo, E.S. (1986) Histological alterations of the liver, gallbladder and common bile duct after papillotomy. *Dig. Dis. Sci.*, **31**, 379S
11. Large, A. (1952) Effect of direct anastomosis of common bile duct to duodenum — experimental study. *Archives of Surgery*, **145**, 450–454
12. Morganstern, L. and Shore, J.M. (1970) Selection of an optimal procedure for decompression of the obstructed common bile duct. *American Journal of Surgery*, **119**, 38–44

13. Safrany, L. (1977) Duodenoscopic sphincterotomy and gallstone removal. *Gastroenterology*, **72**, 338–343
14. Reynolds, M., Luck, S.R. and Raffensperger, J.G. (1985) The valved conduit prevents ascending cholangitis: a follow up. *Journal of Paediatric Surgery*, **20**, 696-702
15. McArthur, M.S. and Longmire, W.P. (Jun.) (1971) Peptic ulcer disease after choledochojejunostomy. *American Journal of Surgery*, **122**, 155–158
16. Roux, C. (1893) Remarques sur 14 gastro-entérostomies, 2 pyloroplasties, sur 1 gastrectomie pour ulcère et 5 gastrectomies pour cancer. *Cong. Franc. Chir. VII*, 394–403
17. Smith, R. (1964) Hepaticojejunostomy: choledochojejunostomy. A method of intrajejunal anastomosis. *British Journal of Surgery*, **51**, 183–186
18. Thorbjarnarson, B. (1975) Bile Duct Injuries. In Surgery of the Biliary Tract. W B Saunders and Co, Philadelphia
19. Warren, K.W. (1973) Prevention and repair of strictures of the extrahepatic bile ducts. *Surgical Clinics of North America*, **53**, 1169–1190
20. Takahashi, T., Ishikawa, Y., Kotoura, Y., Yamamura, T. and Utsunoiya, J. (1988) Follow up studies on various reconstruction methods of the biliary tract including our new method (Roux Y-duodenojejunal anastomosis). *Japanese Journal of Surgery*, **18**, 179–186
21. Kune, G.A. and Sali, A. (1981) Benign Biliary Strictures. Chapter 6 in The Practice of Biliary Surgery, 2nd Edition. Blackwell Scientific Publications, Oxford
22. Gadacz, T.R. (1983) Operations for Strictures of the Bile Ducts. Chapter 31 in Surgery of the Alimentary Tract. Shackelford R.T. and Zuidema G.D., W.B. Saunders and Co, Philadelphia, London, Toronto, Mexico City, Rio de Janeiro, Sydney, Tokyo
23. Bismuth, H., Franco, D., Corette, M.B. and Hepp, J. (1978) Long term results of Roux-en-Y hepaticojejunostomy. *Surgery, Gynaecology and Obstetrics*, **146**, 161–167
24. Blumgart, L.H. (1988) Hilar and intrahepatic biliary-enteric anastomoses. Chapter 70 in Surgery of the Liver and Biliary Tract. Ed. L.H. Blumgart, Churchill Livingstone, Edinburgh, London, Melbourne, New York
25. Summerfield, J.A. (1988) Biliary obstruction is best managed by endoscopists. *GUT*, **29**, 741–745

*(On invitation by S. Bengmark)*

